# Identification of a New Antimicrobial Resistance Gene Provides Fresh Insights Into Pleuromutilin Resistance in *Brachyspira hyodysenteriae*, Aetiological Agent of Swine Dysentery

**DOI:** 10.3389/fmicb.2018.01183

**Published:** 2018-06-19

**Authors:** Roderick M. Card, Emma Stubberfield, Jon Rogers, Javier Nunez-Garcia, Richard J. Ellis, Manal AbuOun, Ben Strugnell, Christopher Teale, Susanna Williamson, Muna F. Anjum

**Affiliations:** ^1^Department of Bacteriology, Animal and Plant Health Agency (APHA), Addlestone, United Kingdom; ^2^APHA Veterinary Investigation Centre Bury St. Edmunds, Bury St Edmunds, United Kingdom; ^3^Central Sequencing Unit, Animal and Plant Health Agency (APHA), Addlestone, United Kingdom; ^4^Farm Post Mortems Ltd., Bishop Auckland, United Kingdom; ^5^APHA Veterinary Investigation Centre Shrewsbury, Shrewsbury, United Kingdom

**Keywords:** *Brachyspira hyodysenteriae*, swine dysentery, antimicrobial resistance, tiamulin, pleuromutilin, antimicrobial resistance gene

## Abstract

*Brachyspira hyodysenteriae* is the aetiological agent of swine dysentery, a globally distributed disease that causes profound economic loss, impedes the free trade and movement of animals, and has significant impact on pig health. Infection is generally treated with antibiotics of which pleuromutilins, such as tiamulin, are widely used for this purpose, but reports of resistance worldwide threaten continued effective control. In *Brachyspira hyodysenteriae* pleuromutilin resistance has been associated with mutations in chromosomal genes encoding ribosome-associated functions, however the dynamics of resistance acquisition are poorly understood, compromising stewardship efforts to preserve pleuromutilin effectiveness. In this study we undertook whole genome sequencing (WGS) and phenotypic susceptibility testing of 34 UK field isolates and 3 control strains to investigate pleuromutilin resistance in *Brachyspira hyodysenteriae*. Genome-wide association studies identified a new pleuromutilin resistance gene, *tva*(A) (*t*iamulin *v*alnemulin *a*ntibiotic resistance), encoding a predicted ABC-F transporter. *In vitro* culture of isolates in the presence of inhibitory or sub-inhibitory concentrations of tiamulin showed that *tva*(A) confers reduced pleuromutilin susceptibility that does not lead to clinical resistance but facilitates the development of higher-level resistance via mutations in genes encoding ribosome-associated functions. Genome sequencing of antibiotic-exposed isolates identified both new and previously described mutations in chromosomal genes associated with reduced pleuromutilin susceptibility, including the 23S rRNA gene and *rplC*, which encodes the L3 ribosomal protein. Interesting three antibiotic-exposed isolates harboured mutations in *fusA*, encoding Elongation Factor G, a gene not previously associated with pleuromutilin resistance. A longitudinal molecular epidemiological examination of two episodes of swine dysentery at the same farm indicated that *tva*(A) contributed to development of tiamulin resistance *in vivo* in a manner consistent with that seen experimentally *in vitro*. The *in vitro* studies further showed that *tva*(A) broadened the mutant selection window and raised the mutant prevention concentration above reported *in vivo* antibiotic concentrations obtained when administered at certain doses. We show how the identification and characterisation of *tva*(A), a new marker for pleuromutilin resistance, provides evidence to inform treatment regimes and reduce the development of resistance to this class of highly important antimicrobial agents.

## Introduction

Pigs are an important source of meat and provide the second highest share of meat consumed worldwide (OECD)^1^. Swine dysentery (SD) is a severe mucohaemorrhagic colitis affecting pigs and is of significant economic, and pig health and welfare importance (Hampson, 2012; Alvarez-Ordóñez et al., 2013). Economic costs of SD can be large, estimated at $115 million to the US swine industry in 1994 and $8.30 to medicate each SD-positive animal in 1990 (Burrough et al., 2013). In the United Kingdom (UK) SD was estimated to cost £4–10 per infected pig in 2012 (Alderton, 2012). These losses arise from reduced feed conversion and weight gain, the high morbidity of disease (up to 90%), costs associated with treatment of clinical disease and metaphylaxis, hygiene measures, and disruption to the trade of pigs (Hampson, 2012; Alvarez-Ordóñez et al., 2013). The disease is distributed worldwide and the classical etiological agent is *Brachyspira hyodysenteriae*, an anaerobic spirochaete which resides in the large intestine of infected pigs. Antibiotic treatment is critical for control of disease on infected units, and is also part of treatment and elimination programmes for SD, especially as no commercial vaccines against SD are available. In most jurisdictions however the number of efficacious antibiotics available to treat SD is severely limited (Hampson, 2012; Kulathunga and Rubin, 2017). For example, in the UK antibiotics authorised for the treatment of SD are limited to the pleuromutilins tiamulin and valnemulin, the macrolides tylosin and tylvalosin, and lincomycin (a lincosamide); although off-label use of other antibiotics (e.g., doxycycline) is permitted under the cascade system, a risk-based decision tree that allows veterinarians to employ clinical judgement to treat an animal with an alternative product when there is no appropriate authorised veterinary medicine available. Tiamulin is the most widely used antibiotic for treatment of SD, due to efficacy towards *B. hyodysenteriae* and relatively short withdrawal periods (van Duijkeren et al., 2014). The World Organisation for Animal Health has classed tiamulin and valnemulin as Veterinary Highly Important Antimicrobial Agents, given their critical importance for the treatment of SD and the lack of alternatives (Anonymous, 2007). In the USA the proposed withdrawal of carbadox (Anonymous, 2014), a compound used to control SD which is already withdrawn from use in the European Union and Canada, and recent recommendations in the European Union to withdraw the indication for oral formulations of tylosin (European Medicines Agency, 2014b) and certain oral lincomycin (European Medicines Agency, 2017) and lincomycin-spectinomycin combinations (European Medicines Agency, 2014a) for treatment of SD caused by *B. hyodysenteriae* would further restrict antimicrobial therapy options available to veterinarians.

A major threat to the effective control of SD is resistance of *B. hyodysenteriae* to pleuromutilins and/or other antibiotics, indeed isolates with reduced susceptibility have been reported in North America, Europe, Japan, and Australia, and the prevalence of resistance appears to be increasing (Karlsson et al., 2002; Lobová et al., 2004; Hidalgo et al., 2009; Pringle et al., 2012; Swedres-Svarm, 2015; Kajiwara et al., 2016; Mirajkar et al., 2016; Mahu et al., 2017; De Luca et al., 2018). Reduced antibiotic susceptibility can lead to suboptimal or ineffective antibiotic treatment, resulting in increased economic impact to producers, adverse effects on pig health and welfare, and development of antibiotic resistance. Furthermore, multidrug resistance has been reported and in some herds *B. hyodysenteriae* has become resistant to all authorised antimicrobials, leaving depopulation and elimination of infection through thorough cleansing and disinfection, and then restocking as the only effective course of action (Hampson, 2012; Strugnell et al., 2013), which has significant cost. Reduced antibiotic susceptibility in *B. hyodysenteriae* has been associated with the presence of *lnu*(C) (lincosamides) (De Luca et al., 2018) and point mutations at specific positions in the 16S rRNA gene (doxycycline), 23S rRNA gene (macrolides, lincosamides, and pleuromutilins) and *rplC*, the gene encoding the L3 ribosomal protein (pleuromutilins) (Karlsson et al., 1999; Pringle et al., 2004, 2007; Hidalgo et al., 2011; Hillen et al., 2014; De Luca et al., 2018). The development of resistance to pleuromutilins in *B. hyodysenteriae* is thought to occur in a stepwise manner both *in vitro* and *in vivo*, suggesting that multiple mutations are required for the emergence of high level resistance (Karlsson et al., 2001; Hidalgo et al., 2011; van Duijkeren et al., 2014), however the dynamics and mechanisms of emergence of resistance to pleuromutilins remain poorly defined. Furthermore *B. hyodysenteriae* isolates with reduced susceptibility to pleuromutilins but without relevant point mutations have been described, while for other mutations there is debate on their role in conferring resistance (Pringle et al., 2004; Hidalgo et al., 2011; Hillen et al., 2014; Mahu et al., 2017). This debate indicates that our understanding is incomplete and suggests that other unidentified mutations and/or genes may be involved in pleuromutilin resistance in *B. hyodysenteriae*.

In this study we have examined the molecular basis for antimicrobial resistance in *B. hyodysenteriae* isolates recovered from pigs in the UK (*n* = 34) and ATCC control strains (*n* = 3) by whole genome sequencing (WGS) and antimicrobial susceptibility testing. Genome-wide association studies were employed to screen for new genes associated with reduced pleuromutilin susceptibility. We additionally investigated mechanisms underlying the emergence of pleuromutilin resistance *in vitro* by sequencing mutant isolates obtained after single exposure of isolates to inhibitory tiamulin concentrations or following repeated culture in sub-inhibitory concentrations. Antibiotic exposure can select for mutational changes conferring resistance to the antimicrobial which has been used, with cross-resistance occurring where those mutations confer resistance to several antimicrobial compounds (Karlsson et al., 1999, 2001; Pringle et al., 2004). We applied the principles of the mutation prevention concentration (MPC) hypothesis, which defines the antibiotic concentration at which mutations giving rise to resistance do not occur (Drlica and Zhao, 2007), when exposing isolates to inhibitory tiamulin concentrations. The MPC has been applied to assess the development of resistance to various antibiotics including quinolones, macrolides, tetracyclines, and pleuromutilins in many bacterial species including *Escherichia coli, Salmonella enterica, Mycoplasma gallisepticum*, and *Staphylococcus aureus* (Randall et al., 2004; Drlica and Zhao, 2007; Ozawa and Asai, 2013; Zhang et al., 2017). Maintaining antibiotic concentrations above the MPC during therapy is thought to help reduce the development of resistance (Drlica and Zhao, 2007) and we related our findings to published tiamulin pharmacokinetic and pharmacodynamic parameters in pigs to help inform veterinary options for the treatment of SD.

## Materials and methods

### Isolates and culture methods

Thirty three UK field isolates of *B. hyodysenteriae* recovered from submissions to the Animal and Plant Health Agency between 2005 and 2013 from 22 pig holdings were used in this study (Table 1). Isolates were derived from diagnostic samples (*n* = 32) or samples collected to assess infection status as part of disease control (*n* = 1). Samples were of three types: excreted faecal samples not collected directly from live pigs (*n* = 20); faeces or intestinal contents collected from dead pigs (*n* = 12, no animals were euthanased specifically for this publication); or rectal swabs (*n* = 1) collected from individual live pigs by veterinary surgeons, which did not require anaesthesia, and was not harmful to the pigs. This sampling strategy is part of the normal veterinary diagnostic investigation of this type of disease on a farm and as such is not for scientific purpose and therefore not covered by the Animal (Scientific Procedures) Act 1986. Sampling which is for the immediate or long term benefit of the individual animal, its immediate cohort or the wider epidemiological group, is covered as an act of veterinary clinical practice within the Veterinary Surgeon's Act 1966. The UK field strain P18A was also included in the panel, which was isolated from a pig with swine dysentery in the late 1970s (Lemcke and Burrows, 1981) and is used as a control for susceptibility testing at APHA (Griffiths et al., 2008). All isolates were recovered from cases of swine dysentery, except BH23 which was isolated from an apparently healthy animal that showed no clinical signs of swine dysentery. At Holdings A and B isolates were recovered on different sampling dates, allowing on-farm disease episodes to be followed; information on tiamulin use was also available for these farms. Additionally, three reference strains were included in this work: B78^T^ (ATCC 27164), B204 (ATCC 31212), and WA1 (ATCC 49526).

**Table 1:** Summary of the 34 field isolates and three reference strains examined in this project, sorted by tiamulin Minimum Inhibitory Concentration (MIC).

**Isolate ID**	**ST**	**Holding**	**Year and month**	**Tiamulin MIC (ECOFF >0.25 mg/L)**	**Valnemulin MIC (ECOFF >0.125 mg/L)**	**23S rRNA**			**L2 protein**	**L3 protein**	***tva*(A)**
						**G2032A**	**G2201T**	**G2535A**	**T50N**	**N148S**	
WA1	26	Reference strain	2000s-n/a	<=0.063	<=0.031						
B204	54	Reference strain	1970s-n/a	<=0.063	<=0.031						
B78T	56	Reference strain	1970s-n/a	<=0.063	<=0.031						
P18A	4	Not known	1970s-n/a	<=0.063	<=0.031						
BH13	88	A	2009-8	<=0.063	<=0.031						
BH26	88	B	2012-1	<=0.063	<=0.031						
BH8	88	C	2008-10	<=0.063	<=0.031						
BH35	91	AR	2012-1	<=0.063	<=0.031						
BH7	239	R1	2008-9	<=0.063	<=0.031						
BH9	88	C	2008-11	0.125	<=0.031						
BH15	8	X	2010-5	0.25	**0.5**			A			*tva*(A)
BH2	8	Z	2005-11	0.25	**0.5**						*tva*(A)
BH3	8	Z	2005-11	0.25	**0.5**						*tva*(A)
BH20	52	CB	2010-12	0.25	**0.25**						*tva*(A)
BH28	88	B	2012-2	0.25	<=0.031					Ser	
BH34	8	CQ	2012-9	**0.5**	**0.5**			A			*tva*(A)
BH16	87	CN	2010-6	**0.5**	0.125		C	A		Ser	
BH14	88	A	2009-10	**0.5**	**1**						*tva*(A)
BH29	88	B	2012-3	**0.5**	**0.5**						*tva*(A)
BH37	240	G	2013-5	**0.5**	**1**			A			*tva*(A)
BH24	52	CM	2011-1	**1**	**0.5**						*tva*(A)
BH6	240	II	2008-7	**1**	**1**			A			*tva*(A)
BH23	167	BF	2010-12	**2**	**1**			A	Asn		*tva*(A)
BH38	52	CP	2013-9	**4**	**4**						*tva*(A)
BH25	8	AB	2011-6	**8**	**4**			A			*tva*(A)
BH27	8	CO	2012-1	**8**	**2**						*tva*(A)
BH17	87	J	2010-7	**8**	**>4**		C	A		Ser	*tva*(A)
BH30	240	H	2012-3	**8**	**2**					Ser	*tva*(A)
BH32	240	H	2012-3	**8**	**4**					Ser	*tva*(A)
BH12	87	J	2009-7	**>8**	**>4**		C	A		Ser	*tva*(A)
BH33	87	K	2012-6	**>8**	**>4**		C	A		Ser	*tva*(A)
BH36	87	O	2013-1	**>8**	**>4**		C	A		Ser	*tva*(A)
BH18	88	A	2010-10	**>8**	**>4**	A					*tva*(A)
BH19	88	A	2010-10	**>8**	**>4**	A					*tva*(A)
BH21	88	A	2010-11	**>8**	**>4**	A					*tva*(A)
BH22	88	A	2010-11	**>8**	**>4**	A					*tva*(A)
BH31	240	H	2012-3	**>8**	**>8**					Ser	*tva*(A)

*Holding of origin is given as an anonymized letter code, together with year and month of sampling. The MICs for tiamulin and valnemulin are shown; bold text indicates MICs above the ECOFF values as given in column headers. The presence of SNPs identified in the 23S rRNA gene and amino acid substitutions in L2 and L3 proteins associated with reduced pleuromutilin susceptibility are indicated; blank, wild-type; ST, Sequence Type. Presence of tva(A) indicated*.

Isolates were cultured on fastidious anaerobe blood agar (FABA) in an anaerobic cabinet (Don Whitley Scientific) in anaerobic gas (10% H_2_, 10% CO_2_, and 80% N_2_) at 38°C for 3–5 days. Broth cultures of *B. hyodysenteriae* were prepared by aseptically picking from the agar surface with a sterile inoculation loop and inoculating into pre-reduced Brain Heart Infusion Broth (BHIB) with 10% Horse Serum (Oxoid or E and O Laboratories Ltd.).

### Susceptibility testing

Minimum Inhibitory Concentrations (MICs) for tiamulin, valnemulin, tylosin, tylvalosin, doxycycline, and lincomycin were determined by broth dilution using VetMIC Brachy plates (National Veterinary Institute, Uppsala, Sweden) (Karlsson et al., 2003). Isolates were plated from stock culture onto FABA plates and sub-cultured twice before testing according to the manufacturer's instructions. Plates were incubated for 4 days at 38°C with shaking at 80 rpm and the MIC was recorded as the lowest concentration of the antimicrobial agent that prevented visible growth. For all samples purity was demonstrated and viable counts (CFU ml^−1^) estimated by creating a 10-fold dilution series in pre-reduced BHIB + 10% FCS and plating on FABA plates. Strain B78^T^ was used as control in each batch of tests (Pringle et al., 2006).

### Selection for resistant mutants at inhibitory concentrations

The isolates selected for these experiments comprised the reference strains B78^T^ and WA1 and 16 field isolates, with different tiamulin MICs spanning ≤ 0.063 to 4 mg/L and different genotypes (STs) (Table S4). Isolates were plated from stock culture onto FABA and sub-cultured twice. For each isolate the growth from four plates was harvested into 10 ml broth culture and incubated overnight at 38°C with shaking at 100 rpm. The McFarland of the broth was determined using a densitometer (Grant Instruments) and 100 μl when then plated onto each of four FABA plates supplemented with dilutions of tiamulin hydrogen fumarate (Sigma-Aldrich, UK) at the MIC as determined by broth dilution and three doubling concentrations above this (Table S4). The purity and CFUml^−1^ of the broth culture was determined by creating a 10-fold dilution series in broth and plating on FABA plates. Plates were incubated for up to 5 days at 38°C. Zones of haemolysis on the antibiotic containing plates indicative of resistant colonies were counted, picked and streaked onto FABA containing tiamulin at the same concentration as the plate picked from. A single CFU was then picked and sub-cultured on FABA with tiamulin until there was sufficient growth to create a stock culture and a cell pellet for DNA extraction. Subsequently, stock cultures of mutant isolates were tested for antibiotic susceptibility as described above. The mutation frequency was calculated as the number of mutants recovered per CFUml^−1^ and the selection index was calculated as the MPC:MIC ratio by dividing the MPC values by the MIC values.

### Selection for resistant mutants at sub-inhibitory concentrations

Ten isolates (Table S6) were plated from stock culture onto FABA and sub-cultured twice. Isolates were then plated onto FABA and a tiamulin MIC Test Strip (Launch Diagnostics, UK) aseptically applied. Subsequently, isolates were sub-cultured twice a week, by harvesting growth along the line of inhibition and re-plating in the presence of a tiamulin MIC Test Strip. As the growth became rich the concentration of tiamulin was increased, using doubling concentrations prepared in sterile discs (Oxoid, UK and Sigma-Aldrich, UK). At points during the experiment a portion of growth was plated onto FABA in the absence of antibiotic, cultured for 3–4 days and used to prepare a stock culture for storage at −80°C and a cell pellet for DNA extraction. Five of the isolates were additionally sub-cultured twice weekly in the absence of tiamulin and stock cultures prepared during the experiment. Selected stock cultures were tested for antibiotic susceptibility as described above.

### Whole genome sequencing and analysis

DNA extracts were prepared from cell pellets using Prepman Ultra (Life Technologies, UK) according the manufacturer's protocol. Nextera XT libraries were prepared for WGS (Illumina, Lesser Chesterford, UK) and sequenced on an Illumina MiSeq platform v2 2x 150 bp paired-end protocol. The raw sequences for each isolate were analysed with the Nullarbour pipeline (version 1.20; Seemann et al.^2^) using the closed genome of WA1 (Bellgard et al., 2009) as reference, and SPAdes version 3.9.0 (Bankevich et al., 2012) and Prokka version 1.11 (Seemann, 2014) for genome assembly and annotation respectively. The published genomes of 41 *B. hyodysenteriae* isolates from swine (Black et al., 2015; La et al., 2016b) were included in this analysis. A maximum likelihood phylogenetic tree using the SNPs located within chromosomal regions present for all the strains was constructed using FastTree (Price et al., 2009). Species were assigned by Kraken (Wood and Salzberg, 2014) (version 0.10.5-beta) and Roary (Page et al., 2015) used to generate gene presence/absence lists. Genome-wide association studies to identify genes having significant association (*p* < 0.05; after Bonferroni correction for multiple tests) with reduced susceptibility to tiamulin and valnemulin were performed using Scoary (Brynildsrud et al., 2016).

Additionally each isolate was analysed using SeqFinder (Anjum et al., 2016), in which the raw sequences were filtered and trimmed using Trimmomatic (Bolger et al., 2014), with the parameters for the minimum quality threshold equal to 20, sliding window equal to 10, and minimum sequence length equal to 36. The raw trimmed and filtered data was mapped onto the genome of the WA1 chromosome (Accession number NC_012225) and plasmid (Accession number NC_012226) (Bellgard et al., 2009) using SMALT (Sanger Institute). The published genomes of 41 *B. hyodysenteriae* isolates from swine (Bellgard et al., 2009; Black et al., 2015) were also mapped to WA1. Single nucleotide polymorphisms (SNPs) with respect to WA1 were calculated using SAMTOOLS software (Li and Durbin, 2009; Li et al., 2009). SNPs were filtered using the quality thresholds of minimum coverage equal to 4, minimum proportion of raw sequences agreeing with the SNP call equal to 80%, and SAMTOOLS SNP quality score >150. Isolate sequence type (ST) was determined by extracting the seven house-keeping genes of the *B. hyodysenteriae* MLST scheme (*adh, alp, est, gdh, glpK, pgm*, and *thi*) (La et al., 2009) and interrogation of the PubMLST database (https://pubmlst.org/brachyspira/). Differences between the genomes of closely related isolates (e.g., parent and mutant isolates) were examined by comparison of the SNPs determined by SeqFinder using custom scripts and by extracting mapped genes of interest for alignment using the Clustal V method in MegAlign (version 11; DNAstar Inc.).

The whole genome sequences and sequence of *tva*(A) from isolate BH14 were deposited in the European Nucleotide Archive under study accession number PRJEB24023.

The presence of SNP mutants associated with reduced susceptibility to antibiotics in the VetMIC Brachy panel was assessed as follows: doxycycline and mutation at G1058 in the 16S rRNA gene (Pringle et al., 2007); tylosin and lincomycin and mutation at A2058 in the 23S rRNA gene (Karlsson et al., 1999; Hidalgo et al., 2011); tylvalosin and a mutation at A2058 and/or A2059 in the 23S rRNA gene (Hidalgo et al., 2011). Reduced susceptibility to tiamulin and valnemulin was assessed using mutations at positions G2032, C2055, G2201, G2447, C2499, C2504, and G2535 in the 23S rRNA gene and with SNPs causing non-synonymous substitutions at amino acids N148 and S149 in the 50S ribosomal protein L3 (Pringle et al., 2004; Hidalgo et al., 2011; Hillen et al., 2014). *E. coli* numbering was used for the 16S and 23S rRNA genes and polypeptide sequences were numbered according to sequence in strain WA1. The correlation of the presence of a SNP with reduced susceptibility was evaluated by two-by-two table analysis (Mackinnon, 2000), where test specificity, sensitivity, and the predictive value of a positive and negative test were calculated using the following criteria: mutant SNP and MIC > ECOFF value were true positive (TP), wild type SNP and MIC ≤ ECOFF value were true negative (TN), mutant SNP but MIC ≤ ECOFF value were false positive (FP), and wild type SNP but MIC > ECOFF value were false negative (FN). The correlation was also evaluated for each antibiotic in this manner for the presence/absence of *tva*(A).

## Results and discussion

### Genome sequencing revealed considerable diversity in UK *B. hyodysenteriae*

**Figure 1:**
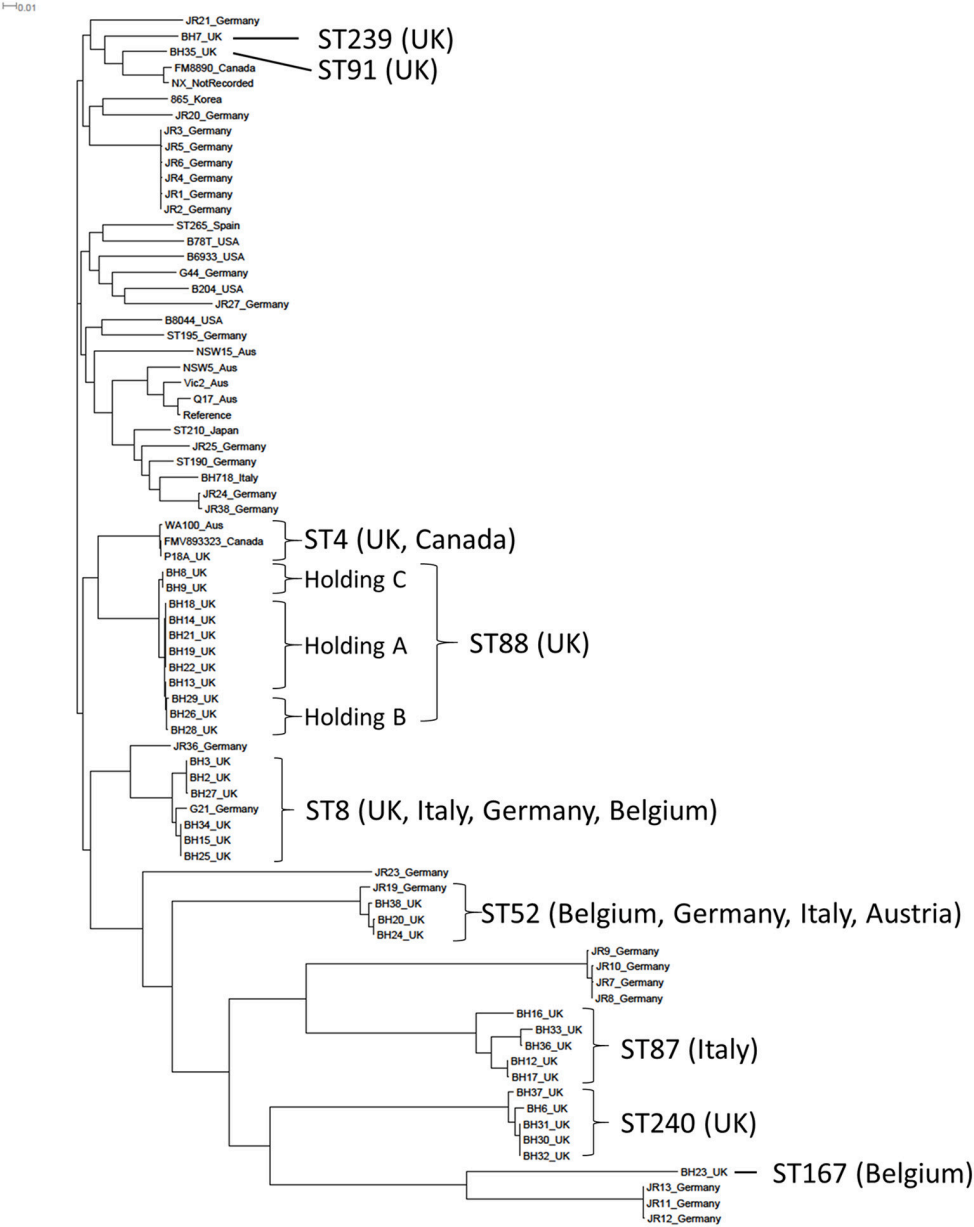
A phylogenetic construction of *Brachyspira hyodysenteriae* isolates using a maximum-likelihood tree. The 34 field isolates sequenced in this project have been included together with 43 published *B. hyodysenteriae* genomes. The Sequence Type (ST) of the 34 isolates sequenced in this study together with countries in which these STs have been described previously is shown.

The 34 UK *B. hyodysenteriae* isolates sequenced for this study were obtained from submissions to APHA between 2005 and 2013, except P18A which was a historical UK strain isolated from a pig with swine dysentery in the late 1970s (Lemcke and Burrows, 1981; Table 1). The genome properties of these isolates, including genome size, GC%, and number of predicted coding sequences were similar to the reference strain WA1 (Bellgard et al., 2009) and other published *B. hyodysenteriae* genomes (Black et al., 2015; La et al., 2016b; De Luca et al., 2018; Table S1). As only 43 *B. hyodysenteriae* genomes have been published to date, the *B. hyodysenteriae* MLST scheme (La et al., 2009) was used to place these UK isolates into a global context. Each UK isolate was assigned to one of eight sequence types (ST) of which two were new variants not represented in the MLST database (https://pubmlst.org/brachyspira/), five had previously been identified in the UK and/or other European countries (Figure 1) and the historical strain P18A (1970s) was ST4 which has been previously described in the UK (NX; 2010s) and Canada (FMV89.3323; 1990s). The Australian isolate WA100 (2010s) was also ST4 by genome analysis, but in the MLST database (La et al., 2009) is classed as ST130 due to a difference in one allele. Importantly, none of the 33 contemporary UK isolates (2005–2013) had STs associated with regions outside Europe, such as North America, Asia and Australia, possibly reflecting pig trading relationships.

A maximum likelihood phylogenetic tree was constructed using core genome SNPs from the WGS of the 34 UK isolates and 43 published *B. hyodysenteriae* genomes (Bellgard et al., 2009; Black et al., 2015; La et al., 2016b; De Luca et al., 2018; Figure 1). Most UK isolates from this study (*n* = 32) formed a sub-cluster that also contained isolates from Germany and the Canadian ST4 isolate. The two UK isolates that did not fall into this group (BH7 and BH35) formed a separate sub-cluster containing a German, a Canadian and a previously sequenced UK isolate. A number of UK and German isolates had considerable diversity in their core genome and formed a distinct sub-cluster (Figure 1). The most distantly related isolates were BH23 from the UK and the weakly haemolytic German isolates JR11-13 (La et al., 2016b). Interestingly, BH23 also had a weak haemolysis phenotype in culture and was isolated from an apparently healthy animal that showed no clinical signs of swine dysentery.

The phylogenetic tree further showed that, while there is considerable diversity in the *B. hyodysenteriae* core genome, the core genome of individual clones remained very stable over prolonged periods of times as demonstrated by the relatively low numbers of SNPs between isolates collected at different times from the same holdings, such as the isolates from Holding A (≤69 SNPs). The diversity and stability of the *B. hyodysenteriae* genome has been noted previously (Black et al., 2015) but these data provide new insight in the farm environment. The phylogenetic tree also gave greater resolution than MLST into the molecular epidemiological investigation of disease episodes at different holdings and identified, for example, three distinct sub-clades from three different holdings within the ST88 branch (Figure 1), including holdings A and B which had known epidemiological links (Strugnell et al., 2013). It is also interesting to note the high degree of core genome conservation in ST4 isolates from three continents collected in different decades, particularly as Australia banned imports of live pigs in the mid-1980s (La et al., 2016a).

### Reduced antibiotic susceptibility can be predicted from genotype

The susceptibility of the 34 field strains and 3 ATCC strains was determined by broth dilution (Karlsson et al., 2003) for tiamulin and valnemulin (Table 1) and for tylsoin, tylvalosin, lincomycin and doxycycline (Table S2). For each antibiotic, an isolate was defined as having reduced susceptibility if the MIC exceeded the environmental cut-off (ECOFF) value (Pringle et al., 2012). The WGS of each isolate was examined for mutations in the 16S rRNA, 23S rRNA, and *rplC* genes associated with resistance to these antibiotics (Table 1). Reduced susceptibility to tylosin, lincomycin, tylvalosin, and doxycycline (i.e., antimicrobial phenotype) correlated well with the presence of relevant mutant SNPs (i.e., genotype), giving good (≥80%) sensitivity, specificity, positive predictive values, and negative predictive values, as calculated using two-by-two table analysis (Table S3) (Mackinnon, 2000), in accordance with previous studies (Karlsson et al., 1999, 2003; Pringle et al., 2007; Hidalgo et al., 2011; Alvarez-Ordóñez et al., 2013; Mahu et al., 2017; De Luca et al., 2018). A new polymorphism (G1058T) associated with reduced doxycycline susceptibility was identified in the 16S rRNA gene of isolates BH6 and BH37.

Correspondence between SNPs and reduced susceptibility to tiamulin and valnemulin was poorer, largely due to the greater number of isolates with reduced susceptibility but no mutation (Table 2), a phenomenon previously noted by others (Pringle et al., 2004; Hidalgo et al., 2011; Hillen et al., 2014; Mahu et al., 2017). To identify new mutations potentially associated with reduced pleuromutilin susceptibility we examined genes encoding the 50S ribosomal proteins L2, L4, and L22 for amino acid substitutions, as they have a possible role in pleuromutilin resistance (Hillen et al., 2014). There was no variation in the L4 amino acid sequence and L22 was also highly conserved. The predicted amino acid sequence of the L2 protein was identical in all but one isolate: BH23 which had a T50N substitution at a conserved threonine residue and a tiamulin MIC of 2 mg/L (Table 1).

**Table 2:** Reduced tiamulin and valnemulin susceptibility predicted by genome sequence based on the presence of mutations in chromosomal genes or the presence of *tva*(A).

	**Tiamulin**		**Valnemulin**	
	**Chromosomal SNPs**	***tva*(A)**	**Chromosomal SNPs**	***tva*(A)**
Sensitivity	77%	**95%**	68%	**100%**
Specificity	**87%**	73%	**83%**	**100%**
Positive predictive value	**89%**	**84%**	**89%**	**100%**
Negative predictive value	72%	**92%**	56%	**100%**
True positive	17	21	17	25
True negative	13	11	10	12
False positive	2	4	2	0
False negative	5	1	8	0

*Isolate genotypes and susceptibilities described in Table 1. Specificity, sensitivity, and the predictive value of a positive and negative test were calculated using two-by-two table analysis (Mackinnon, 2000) with the following criteria: SNP/tva(A) presence and MIC > ECOFF value were true positive (TP), wild type SNP and MIC ≤ ECOFF value were true negative (TN), SNP/tva(A) presence but MIC ≤ ECOFF value were false positive (FP), and wild type SNP but MIC > ECOFF value were false negative (FN). Values ≥ 80% are indicated in bold font*.

### Identification of a new pleuromutilin resistance gene

We next employed a genome-wide association study to search for genes associated with reduced pleuromutilin susceptibility and identified one gene significantly associated with isolates having reduced valnemulin susceptibility (*p* < 0.000003 after Bonferroni correction for multiple tests). Two-by-two table analysis using this gene as a predictor of reduced valnemulin susceptibility gave 100% sensitivity and specificity (Table 2). This gene was also identified when examining reduced susceptibility to tiamulin but the association was not significant (*p* < 0.0606 after Bonferroni correction). However using the gene as a predictor of reduced tiamulin susceptibility gave an improved sensitivity and negative predictive value compared to SNPs only (Table 2); the lower specificity arose because four isolates with a tiamulin MIC at the ECOFF value carried this gene (Table 1). One isolate with reduced tiamulin susceptibility (BH16) did not possess this gene although it did carry three mutations in ribosome-associated genes associated with reduced pleuromutilin susceptibility (Table 1). Of the 14 isolates which had a tiamulin MIC > 2 mg/L, and thus meeting criteria proposed for clinical resistance (Duinhof et al., 2008; Swedres-Svarm, 2015), 12 (86%) carried both the newly identified gene and one or more SNPs associated with reduced pleuromutilin susceptibility (Table 1).

The newly identified 1,518 bp gene encoded a highly conserved 505 amino acid polypeptide in which Pfam analysis (Finn et al., 2016) identified two regions with strong similarity to ABC transporter domains (*E*-values ≤ 3e-10), each containing a Walker A, Walker B, and ABC signature motif, but no transmembrane domain (Figure S1). This structure is found in ATP-binding cassette (ABC) proteins of the ABC-F subfamily (Kerr et al., 2005; Wilson, 2016). Antibiotic resistance ABC-F proteins act as ribosome protection proteins (Sharkey et al., 2016) and have been described in Gram positive bacteria, falling into three main groups according to the resistance phenotypes they confer (Kerr et al., 2005; Sharkey et al., 2016). The newly identified ABC-F gene had an overall amino acid identity of <23% to proteins from these groups and was distantly related in a phylogenetic tree (Figure S2). The new gene was also present in 10 published *B. hyodysenteriae* genomes, but only the Italian isolate BH718 has published pleuromutilin susceptibilities, having tiamulin and valnemulin MICs above the ECOFF (De Luca et al., 2018). Furthermore, a closely related gene (86% amino acid identity) was identified in the *Brachyspira pilosicoli* isolates WesB and B2904 (Figure S2). We have named the *B. hyodysenteriae* ABC-F gene *tva*(A) (*t*iamulin *v*alnemulin *a*ntibiotic resistance) and the *B. pilosicoli* variant *tva*(B). Pleuromutilins target the ribosomal peptidyl transferase centre (Long et al., 2006) and we therefore propose that *tva*(A) may reduce susceptibility to these antibiotics by acting as an ABC-F ribosome protection protein. In future the cloning and overexpression of *tva*(A) in a heterologous system, such as *Escherichia coli*, can be undertaken to examine this further.

**Figure 2:**
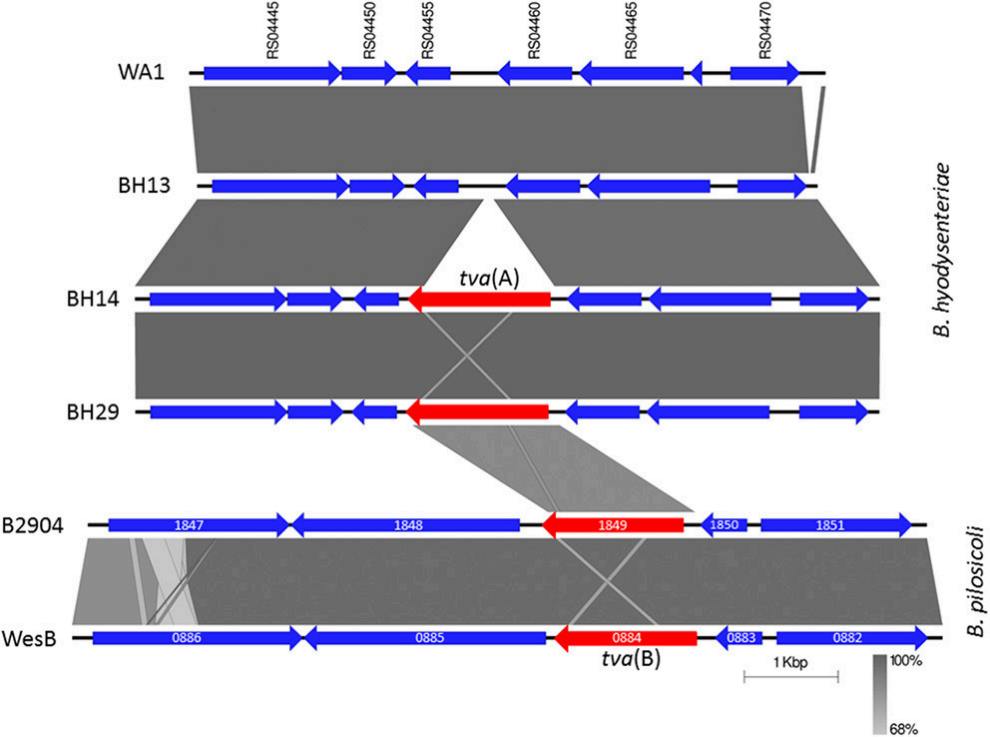
Chromosomal arrangement of genes in *Brachyspira hyodysenteriae* and *Brachyspira pilosicoli* surrounding *tva*(A) and *tva*(B) respectively. *B. hyodysenteriae* genes labelled according to the locus tag in the reference strain WA1 (Accession number NC_012225); *tva*(A) is not present in WA1 and therefore has no locus tag. Genes have been coloured to indicate *tva*(A) and *tva*(B) in red and other genes in blue. WA1 and BH13 were tiamulin susceptible (MIC ≤ 0.063 mg/L). Field isolates BH14 and BH29 were recovered from different holdings, harboured *tva*(A) and had intermediate tiamulin MICs (0.5 mg/L). Also shown is the region surrounding *tva*(B) from *B. pilosicoli* isolates B2904 (Accession number CP003490; locus tag B2904_orf1849) and WesB (Accession number HE793032; locus tag WESB_0884); other genes labelled according to their locus tags. Regions of homology between isolates are shown by grey shading. Image generated using EasyFig (Sullivan et al., 2011).

Resistance mediated by ABC-F proteins in Gram-positive bacteria is often transferable as the genes can reside on mobilisable plasmids. Analysis of the nucleotide region surrounding *tva*(A) for all isolates indicated that it was located on the chromosome and not on the only plasmid present in *B. hyodysenteriae*. Furthermore, the synteny of *tva*(A) was identical in every isolate, being invariably placed between a cell division protein (WA1 locus ID RS04455) and an operon containing an oxidoreductase (RS04460) and an efflux pump of the multi-drug and toxic compound extrusion (MATE) family (RS04465), as shown for two isolates in Figure 2. Although the synteny of *tva*(B) within the two *B. pilosicoli* genomes was identical they had no similarity to *B. hyodysenteriae* synteny (Figure 2). In contrast to the lincomycin resistance gene *lnu*(C) recently reported in *B. hyodysenteriae* (De Luca et al., 2018), no transposon and/or insertion element sequences were identified in the vicinity of *tva*(A). However sequence alignment identified highly conserved motifs upstream and downstream of *tva*(A), absent in isolates without *tva*(A). For example, an AC dinucleotide motif flanked *tva*(A) (Figure S3), which may have been duplicated following insertion and with the subsequent loss of the transposon or insertion sequence, as has recently been described for *mcr-1* in *Moraxella* spp. (AbuOun et al., 2017). Furthermore an inverted repeat flanked *tva*(A) and may indicate a site of recombination (Figure S3). However at present there is insufficient evidence to unambiguously conclude that *tva*(A) is mobilisable but it is interesting to note that the *tva*(A) GC content was not greatly different to the *B. hyodysenteriae* average (27.5% vs. 29.5%).

### The dynamics of tiamulin resistance development *in vitro*

We next examined the development of tiamulin resistance and the significance of *tva*(A) using *in vitro* studies. In one set of experiments 18 isolates were cultured at inhibitory tiamulin concentrations: the MIC as determined by broth dilution and three doubling concentrations above this (Table S4). This approach allowed us to investigate the tiamulin MPC and define the mutant selection window (MSW), which lies between the MIC and the MPC and is the concentration range at which resistant mutants may arise (Drlica and Zhao, 2007). No mutants were observed with the six isolates which did not carry *tva*(A) (Table S4). Fifteen mutants were recovered from five of the six isolates tested which carried *tva*(A) and had a tiamulin MIC between 0.25 and 1 mg/L (Table S4). Of the five tiamulin resistant isolates tested, all harbouring *tva*(A), one mutant was recovered from BH38 (Table S4), the only resistant isolate tested in which no resistance mutations in ribosome-associated genes were identified (Table 1). For 17/18 isolates tested, the MPC was within the tiamulin concentration range used and less than three doublings above the MIC; two mutants were recovered from BH29 at the highest tiamulin concentration used for this isolate (Table S4). All isolates that did not harbour *tva*(A) had MPCs which did not exceed 0.5 mg/L and the selection index (MPC:MIC ratio) was 1, whereas isolates harbouring *tva*(A), and with a tiamulin MIC ≤ 2 mg/L, had MPCs from 0.5 to at least 8 mg/L and a selection index of 1–8 (Table S4). These results indicate that *tva*(A) raises the MPC and widens the MSW. Mutants were only recovered from isolates harbouring *tva(A)* and the geometric mean of mutation frequency was 1.88 × 10^−8^, similar to mutation rates reported for *E. coli* and *S. enterica* exposed to quinolones (Randall et al., 2004; Ozawa and Asai, 2013).

Ten of the 16 mutants were recovered from one isolate (BH20) which we termed as “hypermutable” due to the large numbers of mutants it generated in comparison to other isolates. All purified mutants showed an increase in tiamulin and valnemulin MICs compared to their parent isolate (Table S5) but no increased MICs for tylosin, lincomycin, tylvalosin, and doxycycline. Analysis of the WGS of mutants showed that each had between one and nine new SNPs, absent in the parent isolate, with 10 isolates having only one new SNP (Table S5). Twelve mutants had a SNP in the 23S rRNA gene, of which nine were at positions previously associated with tiamulin resistance (G2032A, C2055T, and C2499T) and three were at new positions (G577A, G1846T, and C1902T), as detailed in Table S5. The polymorphisms at C2055 and C2499 are new mutations associated with tiamulin resistance, as only adenine substitutions at these positions have been reported previously (Pringle et al., 2004). Two mutants had a SNP in *rplC*, one resulting in a S149I amino acid change in the L3 ribosomal protein (described previously Pringle et al., 2004) and the other gave a new amino acid change (S149R). Another mutant had a SNP in the *fusA* gene, encoding Elongation Factor-G (EF-G), which resulted in an A261V substitution at the conserved alanine residue in the G5 box. One mutant isolate had no SNPs in ribosome-associated genes but had a single SNP in a gene encoding ribose-phosphate pyrophosphokinase that resulted in an amino acid substitution (Table S5), but the role of this enzyme in tiamulin resistance is unknown. SNPs not associated with the ribosome were also identified in six other mutants, with most (16/21) located in non-coding regions (Table S5).

In a separate *in vitro* screen for tiamulin resistant mutants, 10 isolates were repeatedly sub-cultured in the presence of sub-inhibitory concentrations of tiamulin for up to 70 sub-cultures, with concentrations increased during this period as growth became rich, similar to earlier studies (Karlsson et al., 1999; Pringle et al., 2004). Five of these isolates were also repeatedly sub-cultured in the absence of tiamulin for the same time period to determine any baseline changes that may occur. Isolates were collected after 30, 45, 60, and/or 70 sub-cultures and tested by broth dilution to determine changes that may have occurred in antibiotic susceptibilities (Table S6). The tiamulin resistant isolate BH30 showed no significant alteration in tiamulin susceptibility after 60 sub-cultures in either the presence or absence of the antibiotic and, following WGS, SNPs were detected in the tiamulin exposed and non-exposed BH30 sub-cultures but none were present in ribosome-associated genes (Table S6). The remaining nine isolates exposed to tiamulin showed from 2 to 5 two-fold increases in tiamulin MIC (Table S6) and a concomitant increase in valnemulin MIC but no alteration in susceptibilities to tylosin, tylvalosin, doxycycline, and lincomycin. Interestingly, for isolates without *tva*(A) (initial MIC ≤ 0.25 mg/L) the MIC post-tiamulin exposure did not exceed 2 mg/L, whereas for isolates harbouring *tva*(A) (initial MIC 0.25–1 mg/L) MICs post-exposure were >2 mg/L, indicating resistance to tiamulin. Genome sequencing showed that 8/9 of these tiamulin-exposed isolates had SNPs in ribosome-associated genes, which were absent in the parent isolate (Table S6). Six isolates had mutations in the 23S rRNA, four at previously described positions (G2032A, C2055T, and G2447T), one at a position also identified in the MPC experiment (G577T) and another at a new position (C2179T). Two isolates (BH15 and BH37) acquired non-synonymous SNPs in the *fusA* gene, which together with the *fusA* mutant from the MPC experiment, provide the first evidence for an association of EF-G with reduced pleuromutilin resistance. EF-G acts during the translocation step of the elongation cycle of bacterial protein synthesis, the step immediately following the peptide-bond formation step which is inhibited by pleuromutilins (Wilson, 2014). Mutations in *fusA* of *Staphylococcus* spp. confer resistance to fusidic acid, an antibiotic that inhibits translocation (Farrell et al., 2011). BH37 additionally acquired a SNP in *rplC* causing as S149I substitution in the L3 protein. The newly identified mutations were not present in any of the UK field isolates analysed or published *B. hyodysenteriae* genomes.

Importantly, sub-culture of the susceptible isolates B78T, BH13, and BH20 in the absence of tiamulin did not alter tiamulin or valnemulin MICs or give rise to mutations in ribosome-associated genes (Table S6). However, for BH14 there was an increase in pleuromutilin MICs both with and without exposure to tiamulin, although no SNPs were identified in ribosome-associated genes in sub-cultured strains from either group, which requires further investigation, as it may represent a subset of strains that can become “naturally” resistant to tiamulin without any exposure possibly through de-repression or up-regulation of some key regulatory genes.

### The role of *tva*(A) in the development of pleuromutilin resistance development

The results provided by the two *in vitro* experiments provide the basis of a hypothesis describing how resistance to pleuromutilin antibiotics develops in *B. hyodysenteriae*, which is summarised in Table 3. The hypothesis derives from the observation that isolates which did not carry *tva*(A) were generally susceptible to tiamulin, and no mutants were recovered from these isolates when exposed to inhibitory tiamulin concentrations. Furthermore, although isolates without *tva*(A) could acquire resistance mutations and consequently reduced pleuromutilin susceptibility following repeated exposure to sub-inhibitory tiamulin concentrations, they did not develop clinical resistance despite prolonged exposure to high tiamulin concentrations (e.g., discs containing 1,000 μg tiamulin). In contrast, generally all isolates with MICs between the ECOFF value and the clinical breakpoint carried *tva*(A); as did four isolates with a tiamulin MIC at the ECOFF (Table 1). Clinically resistant mutants were recovered from 5/6 *tva*(A) isolates following exposure to inhibitory tiamulin concentrations and from the six isolates repeatedly exposed to sub-inhibitory tiamulin concentrations. Therefore our results indicate that *tva*(A) is critical for the development of clinical pleuromutilin resistance and most highly resistant isolates harboured both *tva(A)* and mutations in ribosome-associated genes. Further support is provided by the fact that the same mutation in the 23S rRNA gene increased the tiamulin MIC of an isolate without *tva*(A) to an intermediate level (>0.25 to ≤ 2 mg/L) whereas an isolate with *tva*(A) harbouring these mutations became highly resistant (>2 mg/L); e.g., compare isolates BH13 and BH20 in Table S6.

**Table 3:** Hypothesis for pleuromutilin resistance development in *B. hyodysenteriae*.

**Tiamulin resistance phenotype**		**Resistance genotype**		**Tiamulin inhibitory concentration (Single exposure)**	**Tiamulin sub-inhibitory concentration (Repeated exposure)**	**No antibiotic (Repeated exposure)**
**Category**	**MIC (mg/L)**	**Chromosomal SNPs**	***tva*(A)**			
Susceptible	≤0.25	No	No^a^	No change *(remains susceptible)*	**Becomes intermediate** ***(not resistant)***	No change *(remains susceptible)*
Intermediate	>0.25 to ≤ 2	No	Yes^b^	**Can become resistant**	**Becomes resistant**	No change *(remains intermediate)*
Resistant	>2	Yes^c^	Yes	No change *(remains resistant)*	No change *(remains resistant)*	No change *(remains resistant)*

*Susceptible isolates have tiamulin MICs equal to or less than the ECOFF value (Pringle et al., 2012), resistant isolates exceed the proposed clinical breakpoint (Duinhof et al., 2008; Swedres-Svarm, 2015), and intermediate isolates reside in between*.

a *Four susceptible isolates had an MIC of 0.25 mg/L and harboured tva(A)*.

b *One intermediate isolate had an MIC of 0.5 mg/L, did not carry tva(A) but did harbour chromosomal SNPs associated with resistance*.

c *Two resistant isolates did not harbour known chromosomal SNPs associated with resistance*.

The development of resistance observed *in vitro* during sustained exposure to tiamulin was mirrored *in vivo*, as shown in a longitudinal molecular epidemiological examination of two episodes of swine dysentery at the same farm (Holding A). The same clone was found to be responsible for the two disease episodes, which were separated by 1 year (Figure 1; Table 1). The first episode was treated with tiamulin and the initial isolate (BH13) was susceptible to tiamulin and did not harbour *tva*(A). Isolate BH14, obtained 2 months later during the first episode, had a raised tiamulin MIC of 0.5 mg/L, had only 69 SNPs difference to BH13 in the core genome but now carried *tva*(A). Four isolates recovered a year later (BH18, BH19, BH21, and BH22), during the second disease episode, were clinically resistant and retained *tva*(A) but now carried 1–4 new SNPs not present in BH14, including a G2032A mutation in the 23S rRNA gene (Table 1).

We therefore propose that *tva*(A) confers reduced pleuromutilin susceptibility in *B. hyodysenteriae* that does not lead to clinical resistance but facilitates the development of higher-level resistance via mutations in ribosome-associated genes. This proposed mechanism of resistance development to pleuromutilins aligns with and refines the stepwise manner proposed previously (Karlsson et al., 2001; Hidalgo et al., 2011; van Duijkeren et al., 2014). It is similar to that reported for plasmid-mediated quinolone resistance genes in several species (Jacoby et al., 2014), and likely explains reported contradictions regarding the capability of particular mutations to confer tiamulin resistance.

### Evidence to inform swine dysentery control on-farm

The data we present on the tiamulin MSW and MPC for *B. hyodysenteriae* can also help inform measures designed to prevent the development of resistance on-farm. In the UK, the authorised dosage for tiamulin products for pigs provides for two treatment regimens: high doses at an inclusion level of 100–200 ppm (5–10 mg/kg bodyweight) in feed for 7–10 days to treat clinical swine dysentery caused by *B. hyodysenteriae* and a lower dosage at an inclusion level of 40 ppm (2 mg/kg bodyweight) in feed for 2–4 weeks for the metaphylaxis of swine dysentery (https://www.vmd.defra.gov.uk/ProductInformationDatabase/). Similar regimes are employed in other jurisdictions. There are limited data available for the pharmacokinetics and pharmacodynamics of tiamulin in pigs, but one report presents estimated colon contents concentrations (CCC) for tiamulin following treatment at doses of 38.5 ppm (CCC <1.98 mg/L), 110 ppm (CCC 2.84 mg/L), and 220 ppm (CCC 8.05 mg/L) in feed for 14 days (Burch and Hammer, 2013). Comparison of CCC to the MSW and MPC defined in this work shows that for isolates without *tva*(A), the MPC was exceeded by the CCC obtained at all three doses. Thus either treatment regimen could be expected to deliver sufficient antibiotic to treat infection and prevent emergence of resistance. However, in *tva*(A) positive isolates, the MSW was expanded and the MPC was higher than the CCC obtained with doses at 38.5 ppm and also at 110 ppm for some isolates tested. Thus isolates harbouring *tva*(A) have the potential to acquire high level resistance under these treatment regimens, whereas the higher therapeutic dose should limit development of clinical resistance. Therefore it would be valuable to establish whether the *tva*(A) gene is present or absent in the *B. hyodysenteriae* infecting the pigs. This is particularly important when tiamulin is used at the metaphylactic dose as this would provide an extended opportunity for *B. hyodysenteriae* harbouring *tva*(A) to remain within the MSW, increasing the potential for clinical resistance to develop. Regular determination of isolate susceptibility on farms using tiamulin for metaphylaxis is recommended, particularly if *tva*(A) was present. Use of different licensed antimicrobials presents another option if the *tva*(A) gene is detected, however resistance to these can be common. Increasing the metaphylactic dose to the treatment dose level might limit development of resistance but would require a change in authorisation and further research on issues such as animal safety, environmental impact and other aspects relating to tiamulin use.

The existence and potential mobilisation of *tva*(A) may also prove relevant to human clinical medicine due to the sustained interest in the use of pleuromutilins to treat human bacterial infections; retapamulin was approved for topical use in the USA in 2007 and lefamulin, highly active against multidrug resistant *S. pneumoniae* and *S. aureus*, was recently reported as being in phase III development for systemic use (Eyal et al., 2016).

In conclusion this work has provided new insights into the diversity of *B. hyodysenteriae* genomes, an important aetiological agent for swine dysentery, and demonstrated the utility of WGS approaches for the molecular epidemiological investigation of disease episodes. Reduced antibiotic susceptibility can be confidently predicted from genome sequences and we have described an expanded repertoire of genes and SNPs associated with pleuromutilin resistance. Indeed, the identification of *tva*(A) gives a deeper understanding of the development of resistance to pleuromutilins and provides evidence-based science that can be practically applied on-farm to assist efforts to reduce the development of resistance to this class of highly important veterinary antimicrobial agents.

## Author contributions

MFA, CT, SW: Funding acquisition. MFA, CT, SW, BS, RC: Project conceptualization. RC, JR, ES, RE, MA, JN-G: Investigation, methodology, and data curation. RC, JN, MA, ES: Data analysis and software. RC: Preparation of draft manuscript. All authors reviewed and edited the manuscript.

### Conflict of interest statement

The authors declare that the research was conducted in the absence of any commercial or financial relationships that could be construed as a potential conflict of interest.

## Footnotes

^1^OECD. Meat comsumption (indicator).

^2^Seemann, T., Goncalves da Silva, A., Bulach, D. M., Schultz, M. B., Kwong, J. C., Howden, B. P. (San Francisco: Github). Available online at: https://github.com/tseemann/nullarbor [Accessed: August 03 Aug 2016].

## References

[B1] AbuOun M. Stubberfield E. J. Duggett N. A. Kirchner M. Dormer L. Nunez-Garcia J.. (2017). mcr-1 and mcr-2 variant genes identified in Moraxella species isolated from pigs in Great Britain from 2014 to 2015. J. Antimicrob. Chemother. 72, 2745–2749. 10.1093/jac/dkx286 29091227 PMC5890717

[B2] Alderton S. (2012). Swine dysentery could cost producers £4-10 a pig. Farmer's Weekly 6.

[B3] Alvarez-Ordóñez A. Martínez-Lobo F. J. Arguello H. Carvajal A. Rubio P. (2013). Swine dysentery: aetiology, pathogenicity, determinants of transmission and the fight against the disease. Int. J. Environ. Res. Public Health. 10, 1927–1947. 10.3390/ijerph10051927 23665849 PMC3709357

[B4] Anjum M. F. Duggett N. A. AbuOun M. Randall L. Nunez-Garcia J. Ellis R. J.. (2016). Colistin resistance in Salmonella and *Escherichia coli* isolates from a pig farm in Great Britain. J. Antimicrob. Chemother. 71, 2306–2313. 10.1093/jac/dkw149 27147305

[B5] Anonymous (2007). OIE List of Antimicrobial Agents of Veterinary Importance. OIE International Committee. Paris: World Organisation for Animal Health.

[B6] Anonymous (2014). Carbadox in Medicated Swine Feed; Opportunity for Hearing. Department of Health and Human Services. Federal Register.

[B7] Bankevich A. Nurk S. Antipov D. Gurevich A. A. Dvorkin M. Kulikov A. S.. (2012). SPAdes: a new genome assembly algorithm and its applications to single-cell sequencing. J. Comput. Biol. 19, 455–477. 10.1089/cmb.2012.0021 22506599 PMC3342519

[B8] Bellgard M. I. Wanchanthuek P. La T. Ryan K. Moolhuijzen P. Albertyn Z. J.. (2009). Genome sequence of the pathogenic intestinal spirochete *brachyspira hyodysenteriae* reveals adaptations to its lifestyle in the porcine large intestine. PLoS ONE 4:e4641. 10.1371/journal.pone.0004641 19262690 PMC2650404

[B9] Black M. Moolhuijzen P. Barrero R. La T. Phillips N. Hampson D.. (2015). Analysis of multiple *Brachyspira hyodysenteriae* genomes confirms that the species is relatively conserved but has potentially important strain variation. PLoS ONE 10:e0131050. 10.1371/journal.pone.0131050 26098837 PMC4476648

[B10] Bolger A. M. Lohse M. Usadel B. (2014). Trimmomatic: a flexible trimmer for Illumina sequence data. Bioinformatics 30, 2114–2120. 10.1093/bioinformatics/btu170 24695404 PMC4103590

[B11] Brynildsrud O. Bohlin J. Scheffer L. Eldholm V. (2016). Rapid scoring of genes in microbial pan-genome-wide association studies with Scoary. Genome Biol. 17:238 10.1186/s13059-016-1108-8 27887642 PMC5124306

[B12] Burch D. G. S. Hammer J. M. (2013). Managing Lawsonia and Brachyspira infections Using Pharmacokinetic and Pharmacodynamic Principles. San Diego, CA: American Association of Swine Veterinarians.

[B13] Burrough E. R. McKean J. Schwartz K. J. (2013). Bloody scours (Swine Dysentery): A Costly Re-emerging Disease That Is Preventable. Pork Information Gateway, Clive, IA Available online at: http://porkgateway.org/resource/bloody-scours-swine-dysentery-a-costly-re-emerging-disease-that-is-preventable/

[B14] De Luca S. Nicholson P. Magistrali C. F. García-Martín A. B. Rychener L. Zeeh F.. (2018). Transposon-associated lincosamide resistance lnu(C) gene identified in *Brachyspira hyodysenteriae* ST83. Vet. Microbiol. 214, 51–55. 10.1016/j.vetmic.2017.12.003 29408032

[B15] Drlica K. Zhao X. (2007). Mutant selection window hypothesis updated. Clin. Infect. Dis. 44, 681–688. 10.1086/511642 17278059

[B16] Duinhof T. F. Dierikx C. M. Koene M. G. van Bergen M. A. Mevius D. J. Veldman K. T.. (2008). Multiresistant *Brachyspira hyodysenteriae* in a Dutch sow herd. Tijdschr Diergeneeskd 133, 604–608. 18767301

[B17] Eyal Z. Matzov D. Krupkin M. Paukner S. Riedl R. Rozenberg H.. (2016). A novel pleuromutilin antibacterial compound, its binding mode and selectivity mechanism. Sci. Rep. 6:39004. 10.1038/srep39004 27958389 PMC5154188

[B18] European Medicines Agency (2014a). Linco-Spectin 100 and Associated Names. Veterinary Medicines Division.

[B19] European Medicines Agency (2014b). Veterinary Medicinal Products Containing Tylosin to Be Administered Orally via Feed or the Drinking Water to Pigs. Veterinary Medicines Division London.

[B20] European Medicines Agency (2017). Questions and Answers on Lincocin and Its Associated Names, ed Veterinary Medicines Division London.

[B21] Farrell D. J. Castanheira M. Chopra I. (2011). Characterization of global patterns and the genetics of fusidic acid resistance. Clin. Infect. Dis. 52(Suppl. 7), S487–S492. 10.1093/cid/cir164 21546625

[B22] Finn R. D. Coggill P. Eberhardt R. Y. Eddy S. R. Mistry J. Mitchell A. L.. (2016). The Pfam protein families database: towards a more sustainable future. Nucleic Acids Res. 44, D279–D285. 10.1093/nar/gkv1344 26673716 PMC4702930

[B23] Griffiths I. B. Hunt B. Rogers J. Teale C. Parr J. (2008). Tiamulin activity against *Brachyspira hyodysenteriae*. Vet. Rec. 163:698. 19060324

[B24] Hampson D. J. (2012). Brachyspiral colitis, in Diseases of Swine, ed Zimmerman J. J. Karriker L. A. Ramirez A. Schwartz K. J. Stevenson G. W. (Ames, IA: Wiley-Blackwell), 680–696.

[B25] Hidalgo A. Carvajal A. García-Feliz C. Osorio J. Rubio P. (2009). Antimicrobial susceptibility testing of Spanish field isolates of *Brachyspira hyodysenteriae*. Res. Vet. Sci. 87, 7–12. 10.1016/j.rvsc.2008.10.017 19084246

[B26] Hidalgo Á. Carvajal A. Vester B. Pringle M. Naharro G. Rubio P. (2011). Trends towards lower antimicrobial susceptibility and characterization of acquired resistance among clinical isolates of *Brachyspira hyodysenteriae* in Spain. Antimicrob. Agents Chemother. 55, 3330–3337. 10.1128/AAC.01749-10 21555771 PMC3122432

[B27] Hillen S. Willems H. Herbst W. Rohde J. Reiner G. (2014). Mutations in the 50S ribosomal subunit of *Brachyspira hyodysenteriae* associated with altered minimum inhibitory concentrations of pleuromutilins. Vet. Microbiol. 172, 223–229. 10.1016/j.vetmic.2014.04.021 24948419

[B28] Jacoby G. A. Strahilevitz J. Hooper D. C. (2014). Plasmid-mediated quinolone resistance. Microbiol. Spectr 2, 475–503. 10.1128/microbiolspec.PLAS-0006-2013 25584197 PMC4288778

[B29] Kajiwara K. Kozawa M. Kanazawa T. Uetsuka K. Nakajima H. Adachi Y. (2016). Drug-susceptibility of isolates of *Brachyspira hyodysenteriae* isolated from colonic mucosal specimens of pigs collected from slaughter houses in Japan in 2009. J. Vet. Med. Sci. 78, 517–519. 10.1292/jvms.15-0608 26596637 PMC4829529

[B30] Karlsson M. Fellstrom C. Gunnarsson A. Landen A. Franklin A. (2003). Antimicrobial susceptibility testing of porcine Brachyspira (Serpulina) species isolates. J. Clin. Microbiol. 41, 2596–2604. 10.1128/JCM.41.6.2596-2604.2003 12791886 PMC156507

[B31] Karlsson M. Fellstrom C. Heldtander M. U. Johansson K. E. Franklin A. (1999). Genetic basis of macrolide and lincosamide resistance in Brachyspira (Serpulina) hyodysenteriae. FEMS Microbiol. Lett. 172, 255–260. 10.1111/j.1574-6968.1999.tb13476.x 10188254

[B32] Karlsson M. Gunnarsson A. Franklin A. (2001). Susceptibility to pleuromutilins in Brachyspira (Serpulina) hyodysenteriae. Anim. Health Res. Rev. 2, 59–65. 10.1079/AHRR200118 11708748

[B33] Karlsson M. Oxberry S. L. Hampson D. J. (2002). Antimicrobial susceptibility testing of Australian isolates of *Brachyspira hyodysenteriae* using a new broth dilution method. Vet. Microbiol. 84, 123–133. 10.1016/S0378-1135(01)00444-8 11731165

[B34] Kerr I. D. Reynolds E. D. Cove J. H. (2005). ABC proteins and antibiotic drug resistance: is it all about transport? Biochem. Soc. Trans. 33(Pt 5), 1000–1002. 10.1042/BST0331000 16246031

[B35] Kulathunga D. G. Rubin J. E. (2017). A review of the current state of antimicrobial susceptibility test methods for Brachyspira. Can. J. Microbiol. 63, 465–474. 10.1139/cjm-2016-0756 28324657

[B36] La T. Phillips N. D. Hampson D. J. (2016a). An investigation into the etiological agents of swine dysentery in australian pig herds. PLoS ONE 11:e0167424. 10.1371/journal.pone.0167424 27907102 PMC5131991

[B37] La T. Phillips N. D. Harland B. L. Wanchanthuek P. Bellgard M. I. Hampson D. J. (2009). Multilocus sequence typing as a tool for studying the molecular epidemiology and population structure of *Brachyspira hyodysenteriae*. Vet. Microbiol. 138, 330–338. 10.1016/j.vetmic.2009.03.025 19369014

[B38] La T. Rohde J. Phillips N. D. Hampson D. J. (2016b). Comparison of *Brachyspira hyodysenteriae* isolates recovered from pigs in apparently healthy multiplier herds with isolates from herds with swine dysentery. PLoS ONE 11:e0160362. 10.1371/journal.pone.0160362 27489956 PMC4973917

[B39] Lemcke R. M. Burrows M. R. (1981). A comparative study of spirochaetes from the porcine alimentary tract. J. Hyg. (Lond) 86, 173–182. 10.1017/S0022172400068881 7462601 PMC2133876

[B40] Li H. Durbin R. (2009). Fast and accurate short read alignment with Burrows-Wheeler transform. Bioinformatics 25, 1754–1760. 10.1093/bioinformatics/btp324 19451168 PMC2705234

[B41] Li H. Handsaker B. Wysoker A. Fennell T. Ruan J. Homer N.. (2009). The sequence alignment/map format and SAMtools. Bioinformatics 25, 2078–2079. 10.1093/bioinformatics/btp352 19505943 PMC2723002

[B42] Lobová D. Smola J. Cizek A. (2004). Decreased susceptibility to tiamulin and valnemulin among Czech isolates of *Brachyspira hyodysenteriae*. J. Med. Microbiol. 53(Pt 4), 287–291. 10.1099/jmm.0.05407-0 15017284

[B43] Long K. S. Hansen L. H. Jakobsen L. Vester B. (2006). Interaction of pleuromutilin derivatives with the ribosomal peptidyl transferase center. Antimicrob. Agents Chemother. 50, 1458–1462. 10.1128/AAC.50.4.1458-1462.2006 16569865 PMC1426994

[B44] Mackinnon A. (2000). A spreadsheet for the calculation of comprehensive statistics for the assessment of diagnostic tests and inter-rater agreement. Comput. Biol. Med. 30, 127–134. 10.1016/S0010-4825(00)00006-8 10758228

[B45] Mahu M. Pasmans F. Vranckx K. De Pauw N. Vande Maele L. Vyt P.. (2017). Presence and mechanisms of acquired antimicrobial resistance in Belgian *Brachyspira hyodysenteriae* isolates belonging to different clonal complexes. Vet. Microbiol. 207, 125–132. 10.1016/j.vetmic.2017.05.022 28757011

[B46] Mirajkar N. S. Davies P. R. Gebhart C. J. (2016). Antimicrobial susceptibility patterns of brachyspira species isolated from swine herds in the United States. J. Clin. Microbiol. 54, 2109–2119. 10.1128/JCM.00834-16 27252458 PMC4963479

[B47] Ozawa M. Asai T. (2013). Relationships between mutant prevention concentrations and mutation frequencies against enrofloxacin for avian pathogenic *Escherichia coli* isolates. J. Vet. Med. Sci. 75, 709–713. 10.1292/jvms.12-0131 23328636

[B48] Page A. J. Cummins C. A. Hunt M. Wong V. K. Reuter S. Holden M. T.. (2015). Roary: rapid large-scale prokaryote pan genome analysis. Bioinformatics 31, 3691–3693. 10.1093/bioinformatics/btv421 26198102 PMC4817141

[B49] Price M. N. Dehal P. S. Arkin A. P. (2009). FastTree: computing large minimum evolution trees with profiles instead of a distance matrix. Mol. Biol. Evol. 26, 1641–1650. 10.1093/molbev/msp077 19377059 PMC2693737

[B50] Pringle M. Aarestrup F. M. Bergsjo B. Fossi M. Jouy E. Landen A.. (2006). Quality-control ranges for antimicrobial susceptibility testing by broth dilution of the *Brachyspira hyodysenteriae* type strain (ATCC 27164T). Microb. Drug. Resist. 12, 219–221. 10.1089/mdr.2006.12.219 17002550

[B51] Pringle M. Fellstrom C. Johansson K. E. (2007). Decreased susceptibility to doxycycline associated with a 16S rRNA gene mutation in *Brachyspira hyodysenteriae*. Vet. Microbiol. 123, 245–248. 10.1016/j.vetmic.2007.02.019 17428623

[B52] Pringle M. Landen A. Unnerstad H. E. Molander B. Bengtsson B. (2012). Antimicrobial susceptibility of porcine *Brachyspira hyodysenteriae* and Brachyspira pilosicoli isolated in Sweden between 1990 and 2010. Acta Vet. Scand. 54:54. 10.1186/1751-0147-54-54 22998753 PMC3526423

[B53] Pringle M. Poehlsgaard J. Vester B. Long K. S. (2004). Mutations in ribosomal protein L3 and 23S ribosomal RNA at the peptidyl transferase centre are associated with reduced susceptibility to tiamulin in *Brachyspira* spp. isolates. Mol. Microbiol. 54, 1295–1306. 10.1111/j.1365-2958.2004.04373.x 15554969

[B54] Randall L. P. Cooles S. W. Piddock L. J. Woodward M. J. (2004). Mutant prevention concentrations of ciprofloxacin and enrofloxacin for *Salmonella enterica*. J. Antimicrob. Chemother. 54, 688–691. 10.1093/jac/dkh360 15243029

[B55] Seemann T. (2014). Prokka: rapid prokaryotic genome annotation. Bioinformatics 30, 2068–2069. 10.1093/bioinformatics/btu153 24642063

[B56] Sharkey L. K. Edwards T. A. O'Neill A. J. (2016). ABC-F proteins mediate antibiotic resistance through ribosomal protection. MBio 7:e01975. 10.1128/mBio.01975-15 27006457 PMC4807367

[B57] Strugnell B. W. Ellis R. J. Thomsom J. R. Steventon A. Teale C. Williamson S. M. (2013). Preliminary findings on the use of multi-locus sequence typing (mlst) to investigate outbreaks of swine dysentery in Northern England. Pig J. 68, 82–87.

[B58] Sullivan M. J. Petty N. K. Beatson S. A. (2011). Easyfig: a genome comparison visualizer. Bioinformatics 27, 1009–1010. 10.1093/bioinformatics/btr039 21278367 PMC3065679

[B59] Swedres-Svarm (2015). Consumption of Antibiotics and Occurrence of Antibiotic Resistance in Sweden. Solna/Uppsala: Swedish National Veterinary Institute.

[B60] van Duijkeren E. Greko C. Pringle M. Baptiste K. E. Catry B. Jukes H.. (2014). Pleuromutilins: use in food-producing animals in the European Union, development of resistance and impact on human and animal health. J. Antimicrob. Chemother. 69, 2022–2031. 10.1093/jac/dku123 24793902

[B61] Wilson D. N. (2014). Ribosome-targeting antibiotics and mechanisms of bacterial resistance. Nat. Rev. Microbiol. 12, 35–48. 10.1038/nrmicro3155 24336183

[B62] Wilson D. N. (2016). The ABC of ribosome-related antibiotic resistance. MBio 7:e00598-16. 10.1128/mBio.00598-16 27143393 PMC4959660

[B63] Wood D. E. Salzberg S. L. (2014). Kraken: ultrafast metagenomic sequence classification using exact alignments. Genome Biol. 15:R46. 10.1186/gb-2014-15-3-r46 24580807 PMC4053813

[B64] Zhang N. Ye X. Wu Y. Huang Z. Gu X. Cai Q.. (2017). Determination of the mutant selection window and evaluation of the killing of *Mycoplasma gallisepticum* by danofloxacin, doxycycline, tilmicosin, tylvalosin and valnemulin. PLoS ONE 12:e0169134. 10.1371/journal.pone.0169134 28052123 PMC5215565

