# Supplementary material for: Identification of a New Antimicrobial Resistance Gene Provides Fresh Insights Into Pleuromutilin Resistance in Brachyspira hyodysenteriae, Aetiological Agent of Swine Dysentery
Source: Front Microbiol. 2018 Jun 19;9:1183. doi: 10.3389/fmicb.2018.01183 (PMC6018095; doi:10.3389/fmicb.2018.01183)
Supplement: Supplementary file 7 [file Data_Sheet_1.DOCX]

**Walker A**

MFIKFNKVSFSYDSSDNILNDVSFHIDNSCTAIV**GENGCGKT**TLAKLITG 50

ILKPNSGSIEYSNKNIITAYCDQECINLPDNAENLFYDDSSYSGYLTSIF 100

**ABC signature** **Walker B**

KIDYNYLYRFDT**LSFGE**RKRLQIASALYSNPD**ILVLD**EPTNHIDIECKDI 150

LINVIKRLDCIVIIISHDIDFLDELVEKCIFIRNGNCKIRIGNYTQCRGY 200

EKDEEDYNFSLYEESRKKAKILENRYKKLQNESDAKKSKCGSKRHIDKKD 250

HDAKGKIDAARLAGKDSRLATKAKQAKSLYNNTVMEMESLYTKKREVMDM 300

**Walker A**

EFIGERYKGKFLFYLEAGETKINSIVLRHPELIVKKDSRIGIE**GVNGAGK** 350

**T**SLLNYIIETMYDNSIDKEKIIYIPQDIDRESWNNTFNNIKALDHESLGF 400

**ABC signature** **Walker B**

LMSFVNRLGSNAKSVINSLN**HSPGE**MRKIMLGMAVIKKPY**IIMLD**EPTNH 450

LDIDSIERLEEALISFNCALIIVSHNRNFIKNAVNTLWSIKIEYNYSILD 500

IKNTI 505

**Fig. S1. Predicted amino acid sequence of the *tva*(A) protein.** The Walker A, Walker B and ABC signature motifs are indicated in bold and underlined.
