# Supplementary material for: Identification of a New Antimicrobial Resistance Gene Provides Fresh Insights Into Pleuromutilin Resistance in Brachyspira hyodysenteriae, Aetiological Agent of Swine Dysentery
Source: Front Microbiol. 2018 Jun 19;9:1183. doi: 10.3389/fmicb.2018.01183 (PMC6018095; doi:10.3389/fmicb.2018.01183)
Supplement: Supplementary file 8 [file Data_Sheet_2.DOCX]

**Fig. S2. Phylogenetic tree of representative antibiotic resistance ABC-F proteins**. Protein sequences from Gram positive bacteria (accession numbers given in figure), *tva*(A) from *B. hyodysenteriae* and *tva*(B) from *B. pilosicoli* isolates WesB (Accession number HE793032; locus tag WESB_0884) and B2904 (Accession number CP003490; locus tag B2904_orf1849). One representative sequence for each of the four variants of *tva*(A) identified have been included. The tree was generated using the Clustal V method in MegAlign.

Amino Acid Substitution per 100 residues

0

157.6

20

40

60

80

100

120

140

vga(A)_NC_011605

vga(A)LC_DQ823382

vga(A)_AF117259

vga(A)V_AF186237

vga(C)_CAY33094

vga(E)_CBY88983

vga(B)_AAB95639

vga(D)_ACX92986

mrs(E)_FR751518

msr(D)_AF227521

msr(A)_AB016613

msr(C)_AY004350

optr(A)_KP399637

sal(A)_KC693025

Eat(A)_KF010778

Eat(A)v_KF010779

lsa(A)_AAW30455

lsa(B)_NP_899166

lsa(E)_AFM38048

lsa(C)_AEA37904

BH23_tva

JR38_tva

JR21_tva

BH14_tva

Q17_tva

WESB_tva

B2904_tva
