# Supplementary material for: Identification of a New Antimicrobial Resistance Gene Provides Fresh Insights Into Pleuromutilin Resistance in Brachyspira hyodysenteriae, Aetiological Agent of Swine Dysentery
Source: Front Microbiol. 2018 Jun 19;9:1183. doi: 10.3389/fmicb.2018.01183 (PMC6018095; doi:10.3389/fmicb.2018.01183)
Supplement: Supplementary file 9 [file Data_Sheet_3.DOCX]

**Fig. S3. Alignment of three regions of flanking sequence surrounding *tva*(A).** Representative *Brachyspira hyodysenteriae* isolates without *tva*(A) (n=8) and with *tva*(A) (n=6) included as named in figure. The size of the gap between each region is indicated and nucleotides are numbered according to WA1 sequence (Accession number NC_012225). Sequences divergent from the consensus are in red font. The inverted repeat sequences are indicated by single underlining and fall within the *tva*(A) coding sequence; initiation (ATG) and stop (TAA) codons of *tva*(A) are indicated in bold. The dinucleotide AC repeats unique to *tva*(A) positive isolates, and possibly indicative of an insertion event, are double underlined.

1065447 1065141

| |

WA1 TTTATATGAGAAATAT <-- 290 bp --> AAAAATACTGTATAAATACACATTGTGA

B78T TTTATATGAGAAATAT <-- 289 bp --> AAAAATACTGTATAAATATACATTGTGA

B204 TTTATATGAGAAATAT <-- 289 bp --> AAAAATACTGTATAAATATATATTGTGA

JR7 TTTATATGAGAAATAT <-- 312 bp --> AAAAATACTATATAAATATATATTGTGA

FM8890 TTTATATGAGAAATAT <-- 291 bp --> AAAAATACTGTATAAATATACATTATGA

P18A TTTATATGAGAAATAT <-- 291 bp --> AAAAATACTATATAAATATATATTGTGA

BH13 TTTATATGAGAAATAT <-- 291 bp --> AAAAATACTGTATAAATATATATTGTGA

BH16 TTTATATGAGAAATAT <-- 312 bp --> AAAAATACTATATAAATATATATTGTGA

BH14 TTTATAACAGCAATAT <76 bp> GGTTAATAT**ATG**TTTATAAAA <1493 bp> AAAAACACTATA**TAA**ACATATATTATGA

BH33 TTTATAACAGCAGTAT <76 bp> GGTTAATAT**ATG**TTTATAAAA <1493 bp> AAAAACACTATA**TAA**ACATATATTATGA

BH23 TTTATAACAGCAATAT <76 bp> GGTTAATAT**ATG**TTTATAAAA <1493 bp> AAAAACACTATA**TAA**ACATATATTATGA

JR11 TTTATAACAGCAATAT <76 bp> GGTTAATAT**ATG**TTTATAAAA <1493 bp> AAAAACACTATA**TAA**ACATATATTATGA

JR38 TTTATAACAGCAATAT <76 bp> GGTTAATAT**ATG**TTTATAAAA <1493 bp> AAAAACACTATA**TAA**ACATATATTATGA

JR21 TTTATAACAGCAATAT <76 bp> GG-TAATAT**ATG**TTTATAAAA <1493 bp> AAAAACACTATA**TAA**ATATATATTATGA
